# Re-Imagining the Future: Repetition Decreases Hippocampal Involvement in Future Simulation

**DOI:** 10.1371/journal.pone.0069596

**Published:** 2013-07-23

**Authors:** Valerie van Mulukom, Daniel L. Schacter, Michael C. Corballis, Donna Rose Addis

**Affiliations:** 1 School of Psychology, The University of Auckland, Auckland, New Zealand; 2 Centre for Brain Research, The University of Auckland, Auckland, New Zealand; 3 Department of Psychology, Harvard University, Cambridge, Massachusetts, United States of America; University of Texas at Dallas, United States of America

## Abstract

Imagining or simulating future events has been shown to activate the anterior right hippocampus (RHC) more than remembering past events does. One fundamental difference between simulation and memory is that imagining future scenarios requires a more extensive constructive process than remembering past experiences does. Indeed, studies in which this constructive element is reduced or eliminated by “pre-imagining” events in a prior session do not report differential RHC activity during simulation. In this fMRI study, we examined the effects of repeatedly simulating an event on neural activity. During scanning, participants imagined 60 future events; each event was simulated three times. Activation in the RHC showed a significant linear decrease across repetitions, as did other neural regions typically associated with simulation. Importantly, such decreases in activation could not be explained by non-specific linear time-dependent effects, with no reductions in activity evident for the control task across similar time intervals. Moreover, the anterior RHC exhibited significant functional connectivity with the whole-brain network during the first, but not second and third simulations of future events. There was also evidence of a linear increase in activity across repetitions in right ventral precuneus, right posterior cingulate and left anterior prefrontal cortex, which may reflect source recognition and retrieval of internally generated contextual details. Overall, our findings demonstrate that repeatedly imagining future events has a decremental effect on activation of the hippocampus and many other regions engaged by the initial construction of the simulation, possibly reflecting the decreasing novelty of simulations across repetitions, and therefore is an important consideration in the design of future studies examining simulation.

## Introduction

Remembering past events and imagining future events recruits a subset of regions of the default network [1], including medial prefrontal and parietal cortices, and the medial temporal lobes (MTL) [2], [3], [4]. Together, these regions have also been described as the ‘core’ network, reflecting the overlapping contributions of these regions to memory and simulation [5], [6]. However, imagining future events has been shown to activate certain regions of this core network, including the anterior right hippocampus (RHC), significantly more than remembering past events [2], [7], [8]. We have argued that while both remembering and imagining employ access to episodic memory details, which form the ‘building blocks’ of events, future simulation also requires the flexible integration of details extracted from various memories into a coherent representation [5], [9]. This process, termed ‘detail recombination’, likely requires additional processing supported by core network regions such as the anterior hippocampus [10].

In contrast, some studies report that remembering past events engages core network regions more than imagining future events [6], [11], [12]. While these findings appear to speak against the idea that, relative to remembering, future simulation requires additional neural resources to support more extensive constructive processes [2], [5], it is notable that the paradigms used in these studies did not require the online construction of imagined events in the scanner. Thus, one way to reconcile these seemingly contradictory findings is to suppose that while the construction of an event draws on hippocampal resources, imagining “pre-constructed” events may not do so to the same degree. We have observed previously that hippocampal activity reduces across the duration of a future simulation trial, with maximal activity evident in the initial moments of event construction [2]. When creating an event ‘from scratch’, the novel binding of details requires more constructive processing than when these details have been linked previously. Indeed, a number of studies have linked robust hippocampal activity with the recombining of familiar elements to form novel associations [13], [14]. Similarly, events that are more demanding to imagine, such as improbable future events, are associated with heightened responses in the anterior RHC [15].

To directly investigate the effects of repetition on simulation-related neural activity, participants repeatedly simulated future events in this study. In particular, we predicted that anterior RHC activity and its connectivity with other structures in the core network would decrease with repetitions, reflecting decreased constructive processing during simulation.

## Materials and Methods

### Participants

Ethics approval was obtained from the Human Ethics Participants Committee at The University of Auckland, New Zealand. Twenty-five healthy, right-handed adults provided their written consent to participate in this study. All participants were fluent in English, had no history of neurologic or psychiatric conditions or use of psychotropic medications, had no fMRI contraindications (e.g., ferromagnetic implants), and had not participated in our previous fMRI studies on future simulation. Five participants were excluded due to excessive movement or insufficient responses; data from 20 participants (nine males; range, 18–30 years) are presented.

### Procedure

We adapted the episodic recombination paradigm [10] to include a repetition-suppression manipulation [16]. The experiment consisted of three phases: A pre-scan session in which memories were recalled, a scan session in which participants imagined future events, and a post-scan interview in which participants were interviewed about the content and features of their imaginings.

#### Pre-scan session

**Figure 1:**
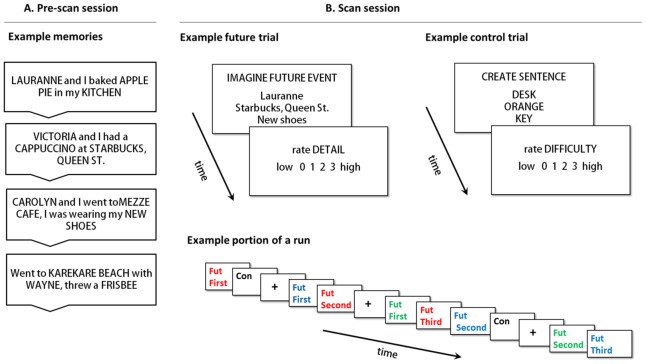
The future event simulation paradigm. (A) Pre-scan session: Participants recalled memories and identified a unique person, location and object in each. (B) Scan session: Participants imagined future events containing the three recombined memory details, and subsequently rated these simulations for detail. Participants also completed control trials, during which three common nouns are incorporated in the sentence “X is bigger than Y is bigger than Z”, followed by a difficulty rating. Runs also contained fixation trials. The sequence of trials from a portion of an example run is also provided in the bottom panel of (B): Repetitions of any one future event were separated by a variable number of intervening trials (other future event trials, control trials and fixation trials).

Participants recalled 100 episodic events from the past ten years. For each memory, a person, location and object were identified and described in a few words or less, with the restriction that these details could not be duplicated across events. Details were randomly recombined into new person-location-object sets where all three details came from different memories (see Figure 1).

#### Scan session

Approximately one week later (*M* = 8.05 days, *SD* = 1.73 days), participants completed the scanning session. Prior to entering the scanner, a practice session was completed to familiarize the participants with the tasks and to allow time for questions. The scanning session consisted of a structural scan (10 mins) and five 12-min functional runs (60 mins). Sixty recombined detail sets were presented during magnetic resonance imaging (MRI); each was shown three times across the scanning session. During the *First* presentation (8*s*), the instruction “imagine future” was provided and participants imagined a novel event incorporating the three details that might occur in the next 5 years. Once participants had an event in mind, they made a button-press and continued imagining until a four-point rating scale for imagined detail (0 = vague; 3 = vivid) was presented (4*s*). The *Second* and *Third* presentation of each set occurred within the same run after an average interval of 93.57 seconds (*SD = *26.00*s*). The duration was pseudo-randomized to avoid regularity, and included one to three future trials and a variable number of null and control trials. Participants were re-presented with an already viewed detail set and instructed to “re-imagine” the original simulation. Participants were encouraged to allow the previously constructed event come to mind, but to refrain from radically changing the simulation (such as progressing the event in time). It was considered acceptable if details in the event became ‘clearer’ over presentations (i.e., better or more easily visualized) as long as the event itself was not changed in any major way (e.g., a change in location, a change in people present, etc.) Participants also completed 60 trials of a size judgement task (adapted from [10]). This control task was designed to include similar elements to the future tasks, namely the presentation of the task instruction and three stimuli words on the screen, mental imagery and the formation of an integrated representation. In this task, participants were presented with a set of three nouns taken from Clark and Paivio’s extended norms [17], and were required to visualize the stimuli and incorporate them into a sentence of the form “X is bigger than Y is bigger than Z”, thus performing a relative size judgment task. Nouns were all rated highly familiar (*M* = 5.69), imageable (*M* = 5.55) and concrete (*M* = 6.94) [17]. Participants then rated the size judgment task for difficulty (4*s*) on a four-point scale (0 = not difficult; 3 = extremely difficult), included to control for the act of making a rating in the future task. One fifth of total scan time comprised jittered fixation-cross trials (4–16*s*) interspersed through the five runs (each 720*s*), as determined using Optseq2 [18].

#### Post-scan session

Immediately following scanning, participants described each event that was imagined in the MRI, estimated the date of future occurrence, rated event novelty relative to previous thoughts and experiences (0 = novel; 3 = identical) and the consistency of the simulation across repetitions (0 = different; 3 = identical; note that consistency referred to event content rather than clarity of the representation, as described above).

### MRI Acquisition

Anatomical data were acquired on a Siemens 1.5T Avanto MRI scanner using an MP-RAGE sequence. Functional scans (25 coronal-oblique interleaved 5 mm slices) were collected perpendicular to the long axis of the hippocampus with a T2*-weighted EPI sequence (TR = 2000 *ms*, TE = 23 *ms,* FOV = 200 mm, flip angle = 90^o^). Stimuli were projected onto a screen reflected into a mirror within the head coil. E-Prime software (Psychology Software Tools Inc.) was used to present stimuli and collect responses made on a 4-button MR-compatible button box.

### MRI Preprocessing

Imaging data were preprocessed and analyzed using SPM8 (Wellcome Trust Centre for Neuroimaging, London). Standard preprocessing included slice-timing correction, rigid-body motion correction and unwarping, spatial normalization to the Montreal Neurological Institute (MNI) template (using normalization parameters derived during segmentation; resampled at 2 mm^3^), spatial smoothing (8 mm full-width half-maximum Gaussian kernel), and high-pass filtering (128 *s* cut-off). One participant’s data contained slight movement artefacts (<6 mm) in 4.7% of TRs. ArtRepair software (http://web.mit.edu/swg/software.htm) was used to repair slice artifacts in raw functional images before preprocessing, and volume artifacts after realignment but before estimation. Each event was modeled by SPM’s canonical hemodynamic response function, applied at stimulus onset.

### fMRI Contrast Analyses

Fixed-effects subject-level models consisted of four regressors of interest: *First, Second,* and *Third* future conditions, *Control* condition. Two regressors of no interest (excluded trials; ratings phase) were also modelled. The future condition regressors included trials for which a reaction time (RT) was collected on all three repetitions (94.65% of all trials). We also used additional RT criteria to exclude trials on which accidental button presses were made (3.6% of all trials). For the *First* condition, we excluded trials where RT was less than 2 seconds, in line with previous research indicating that it takes participants approximately 2 seconds to read a screen with instructions and cue words on the first presentation [2]. For repeated imaginings, we excluded any trials where RT was more than two standard deviations lower than the mean RT for that condition and therefore trials faster than 374 *ms* for *Second* and 15 *ms* for *Third* were excluded. Trials for which simulations did not comply with task instructions were also excluded, according to the following criteria: simulations rated (at post-scan) as “identical” to previous thoughts/experiences (0.9% of all trials); simulations rated as “different” with respect to consistency over presentations (0.3% of all trials). Given that for some trials multiple exclusionary criteria applied, in all 91.42% of the original trials were entered into the analyses.

A random-effects flexible factorial model with two factors, *condition* and *subject,* was computed using contrast images for conditions (relative to the implicit baseline) from the fixed-effects models. Following an omnibus *F* test to assess the effect of condition, we examined the regions engaged during future simulation relative to the control task with the contrast *Future*(First, Second, Third)>*Control*. We also computed two contrasts testing for linear trends: *First*>*Second>Third* (c = [1 0–1]) and *Third*>*Second>First* (c = [−1 0 1]). Note that these contrast weights are recommended for testing linear trends over three conditions [19]. We tested for non-linear effects using two quadratic contrasts (c = [−1 2 −1] and [1 −2 1]).

We computed additional analyses to determine whether any changes in signal across repetitions were the result of non-specific linear time-dependent effects, that is, increases or decreases in signal unrelated to the repetition manipulation that occur across the time window between the *First* and *Third* future event trials. While we had controlled for low frequency signal drift across the duration of the entire run with a high-pass filter (128 *s* cut-off), we computed a new analysis to control for any non-specific linear time-dependent effects occurring across the time interval separating the first and third future trials (*M* = 93.57 *s*, *SD = *26.00 *s*). In order to model the change in signal over this time window, we divided our control trials into pairs separated by a time interval similar to that between the *First* and *Third* future trials (*M* = 89.31 *s*, *SD* = 48.29 *s*; these intervals were not significantly different from those for the future trials, *t_38_* = .49, *p* = .63). We re-ran the fixed-effects and random-effects flexible factorial models to include the *time_1_* and *time_2_ Control* conditions. A *repetition* × *condition* interaction analysis was computed to identify regions with significant repetition effects (increases or decreases) for the *Future* but not the *Control* conditions.

To investigate whether decreasing reaction times were related to changes in neural activity across repetitions, we entered reaction times as a parametric modulation regressor in the fixed-effects model, producing contrast images for each condition of interest that were independent of the effect of reaction time. These contrast images were entered into the random-effects flexible factorial model and we re-computed our *First*>*Second>Third* and *Third*> *Second>First* contrasts, controlling for reaction time. We also used these fixed-effects models to run a random-effects parametric modulation analysis to identify regions in which neural activity correlated with reaction time. A contrast image of the parametric modulation effect from each participant’s fixed effects model was entered into a random-effects one-sample *t*-test to identify regions where activity was significantly correlated with reaction times at the group level.

A correction for multiple comparisons was applied to all contrasts (*p_FWE_*<.05). Peak MNI coordinates were transformed into Talairach space for localization using a stereotactic atlas [20]. All coordinates are reported in MNI space. For descriptive purposes, percent signal change data were extracted from 2 mm spheres centred on peak voxels. Masks were created in MarsBaR [21] and the REX toolbox (http://web.mit.edu/swg/software.htm) was used to extract and rescale beta values to percent signal change.

We also computed a laterality index for hippocampal activity, using the Laterality Index (LI) toolbox [22] and an anatomical AAL atlas mask of the bilateral hippocampus from the WFU PickAtlas Tool (http://fmri.wfubmc.edu/software/PickAtlas). The LI for the *Future*>*Control* and *First*>*Second>Third* contrast images was determined using a bootstrap analysis that determines the laterality of activity using a sum of voxel values at different thresholds. LI values range from −1 (extreme right) to +1 (extreme left).

### Functional Connectivity Analyses

We used partial least squares (PLS), a covariance-based multivariate technique [23], [24], to examine whether the connectivity of the anterior RHC with other brain regions also differs according to repetition. A seed analysis was computed using maximal signal extracted from the peak RHC voxel in contrast of *Future*>*Control* (see *Results*); this contrast was used for voxel selection so as not to bias activity to show decreasing connectivity across repetitions. Correlations between activity in this seed voxel and all other voxels were computed for each condition (across an 18*s* trial window) across participants. The resulting correlation maps were stacked and analyzed with singular value decomposition. We utilized a non-rotated version of PLS, specifying two *a priori* contrasts: (1) stronger RHC connectivity during *First* relative to *Second* and *Third*; and (2) stronger RHC connectivity during *First* and *Second* relative to *Third*. For each contrast, a latent variable was produced, comprising a singular value (indicating the amount of covariance for which the LV accounts), a linear contrast between the seeds and the conditions (coding for the effect depicted by voxels), and a singular image of voxel weights or “saliences” (akin to a component loadings in principle components analysis) that are proportional to the covariance of activity with the linear contrast.

The significance of each LV was determined using permutation testing in which each participant’s data were randomly reassigned to experimental conditions and the PLS analysis recomputed to obtain a new singular value for each reordering. This permutation procedure was done 500 times, and thus significance reflects the number of times the singular value from the permuted data exceed the original singular value (*p≤*.05). Because whole-brain patterns are assessed in one analytic step, corrections for multiple comparisons are not required. The reliability of voxel saliences was determined using bootstrap estimation of the SE: participants were randomly resampled with replacement, the PLS analysis was rerun and new saliences were determined. After 300 iterations, the SE of the salience was computed. Clusters of five or more voxels in which bootstrap ratios were greater than ±5 (*p*<.0001) were considered reliable.

## Results

### Behavioral Results

Behavioral data are presented in Table 1. A repeated-measures ANOVA showed that RTs significantly decreased over repetitions *F*_(1.15,21.92)_ = 120.85, *p*<.001, with differences between all future conditions (*p_Bonferroni_*<.002). A Friedman test showed that detail ratings increased across repetitions (χ^2^_(2)_ = 33.60, *p*<.001), and Wilcoxon Signed-Rank Tests confirmed that detail ratings differed between all future conditions (Z scores <−3.06; *p*-values ≤.002). The average estimated date of future simulated events was 1.84 years from the present. Simulations were rated as having minimal similarity to previous thoughts and past experiences, and highly consistent over repetitions.

**Table 1:** Mean reaction times, detail ratings, and post-scan ratings of future events.

Measure	Mean scores (SD) according to condition		
	*First*	*Second*	*Third*
Reaction time (*s*)	4.37 (.92)	2.80 (1.21)	2.55 (1.27)
Detail of simulation^†^	1.39 (.38)	2.04 (.29)	2.26 (.29)
	Mean scores (SD)		
Temporal distance of event (years)	1.84 (.73)		
Similarity of event to previous experiences^†^	0.36 (.19)		
Similarity of event to previous thoughts^†^	0.11 (.12)		
Consistency of event across repetitions^†^	2.75 (.17)		

Note: ^†^Participant ratings made using a four-point rating scale, ranging from 0 (low) to 3 (high).

### fMRI Contrast Results

All contrast results presented here were computed within the random-effects flexible factorial model. In order to identify the regions associated with the construction of future simulations, we computed a contrast of *Future*>*Control.* This contrast replicated previous findings of simulation-related activity in medial prefrontal and parietal cortex, lateral temporal cortex and MTL (a cluster which extended into the RHC, *xyz* 32 −16 −18; see Table 2).

**Table 2:** Regions evident in *Future*>*Control* contrast analysis.

Brain Region	MNI co-ordinates			Z-score
	x	y	z	
Future>Control^*^				
L Posterior Cingulate Gyrus/Cingulate Gyrus (BA 31)^†^	−6	−62	24	*Infinite*
L Medial Frontal Gyrus (BA 10/11)^†^	−2	58	−6	*Infinite*
R Middle Temporal Gyrus (BA 21)	58	−6	−18	7.75
L Middle Temporal Gyrus (BA 39)	−42	−70	30	7.59
R Parahippocampal Gyrus (BA 36)	24	−38	−10	7.08
L Superior Frontal Gyrus (BA 9)	−18	34	38	6.92
R Supramarginal Gyrus (BA 40)	52	−53	22	6.89
L Inferior Temporal Gyrus	−62	−10	−20	6.66
R Middle Frontal Gyrus	20	34	44	6.47
L Parahippocampal Gyrus	−24	−40	−8	5.61

*Note:* All activations evident at a height threshold of *p_FWE_* <.05; for brevity, only those clusters with more than 100 voxels are reported. Only the maximal peak voxel of each cluster is reported. BA = Brodmann area; L = left; R = right; ^†^Cluster extends bilaterally. ^*^All regions in future>control contrast were also evident in an *F*-test assessing the main effect of condition.

**Figure 2:**
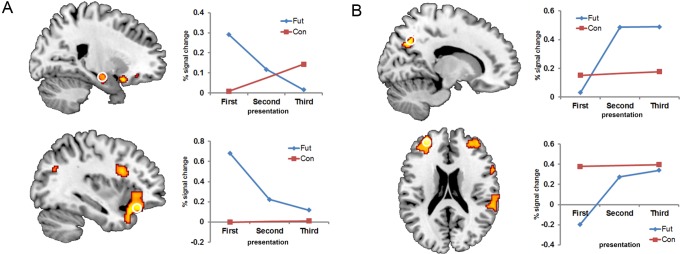
Results from contrast analyses. (A) Regions from the interaction analysis in which fMRI signal decreased across repetitions for the future condition only: right hippocampus (*xyz* 22 −10 −14, top panel) and left inferior frontal gyrus (−36 28 −16, bottom panel), and associated percent signal change data for future and control conditions (First* =  First*_Future_ and *time1*_Control_ conditions; Second = *Second*_Future_; Third =  *Third*_Future_ and *time2*_Control_ conditions). These regions were also evident in the contrast of *First*>*Second>Third* (see Table 3). (B) Regions from the interaction analysis in which fMRI signal increased across repetitions for the future condition only: right precuneus (14 −64 38, top panel) and left anterior prefrontal cortex (−30 52 20, bottom panel) with associated percent signal change data. These regions were also evident in the contrast of *Third*>*Second>First* (see Table 3). Activity is shown at *p_uncorrected_* <.0001 overlaid on a standard anatomical template; all peak activations survived a corrected threshold of *p_FWE_* <.05; see Table 3. Note that error bars are not included as these plots are for descriptive purposes only [54].

To further explore whether activation of these simulation-related regions were modulated by repetition, we ran a set of linear and quadratic contrasts. The contrast of *First*>*Second>Third* revealed a profile of decreasing activity across repetitions in MTL regions, including a cluster in the anterior RHC (that extended into the right amygdala, *xyz* 16 −6 −18 and parahippocampal gyrus, 34 −28 −18), the left amygdala, bilateral inferior frontal gyri, and left medial prefrontal and posterior cingulate cortices (Table 3, Figure 2A). LI results confirmed that hippocampal activity during both this contrast and the *Future>Control* contrast was strongly right-lateralized (LIs: *Future*>*Control*, −0.71; *First*>*Second>Third*, −0.51). To further explore the nature of this linear decrease in RHC activity across repetitions, we computed whole-brain contrasts *First*>*Second* and *Second*>*Third.* Both contrasts revealed activation of the same regions of the RHC activity (*xyz* 32 −16 −18), albeit at a lower threshold (*p_uncorrected_* <.001) than when the entire linear trend was assessed, but still in line with the finding that the right hippocampus exhibits a linear rather than non-linear decrease over repetitions.

**Table 3:** Regions evident in repetition contrast analyses.

Brain Region	MNI co-ordinates			Z-score
	x	y	z	
*First>Second>Third*				
L Medial Frontal Gyrus (BA 10)	−4	58	−6	7.76
L Caudate (Head)^*^	−6	12	−6	7.06
L Inferior Frontal Gyrus (BA 47)	−38	30	−16	6.42
R Inferior Frontal Gyrus (BA 47)	30	32	−12	6.41
R Hippocampus^§^	32	−16	−18	6.32
L Middle Temporal Gyrus (BA 39)	−42	−70	30	6.32
L Posterior Cingulate/Retrosplenial Cortex (BA 31)^∫^	−4	−56	22	6.31
R Superior Temporal Gyrus^*†^	40	18	−30	6.27
L Middle Temporal Gyrus (BA 21)^*^	−60	−10	−16	6.01
L Medial Frontal Gyrus (BA 10)^†^	−12	64	12	5.97
L Amygdala	−18	−6	−14	5.61
R Middle Frontal Gyrus (BA 9)	40	14	28	5.57
L Medial Frontal Gyrus (BA 8)^*§^	−14	34	46	5.47
*Third>Second>First*				
R Inferior Frontal Gyrus (BA 44)^¥^	58	14	10	7.39
L Superior Frontal Gyrus (BA 10)	−30	54	20	6.98
R Inferior Parietal Lobule (BA 40)^¥^	56	−44	38	6.85
R Precuneus (BA 7)	14	−66	36	6.62
L Cingulate Gyrus (BA 23)	−2	−26	28	6.09
R Middle Frontal Gyrus (BA 10)^†^	44	50	8	6.02

*Note:* All activations evident at a height threshold of *P_FWE_* <.05; for brevity, only those clusters with more than 100 voxels are reported. Only the maximal peak voxel of each cluster is reported. BA = Brodmann area; L = left; R = right; ^¥^Cluster also exhibits a quadratic effect.^ †^Cluster not evident in the interaction analysis (controlling for non-specific linear time-dependent effects). ^*^Cluster not evident when controlling for reaction time. ^§ ∫^Peak voxel for cluster is shifted to an adjacent voxel when controlling for time-dependent (^§^) and reaction time (^∫^) effects.

The opposite linear contrast, *Third*>*Second>First*, demonstrated that activity in left anterior and right ventrolateral prefrontal cortex, right inferior parietal lobule, right posterior cingulate gyrus and right ventral precuneus increased with repetition (Table 3, Figure 2B). Importantly, no MTL region showed increasing activation across repetitions. Although some regions are evident in both linear contrasts (*First*>*Second*>*Third* and *Third*>*Second*>*First*), such as the left posterior cingulate, right inferior and middle frontal gyri, it is important to note that different subregions of these neural structures exhibit opposite linear effects, as indicated by the different Brodmann area labels in Table 3; there was in fact no overlap between the statistical maps resulting from these two linear contrasts.

To test for possible nonlinear effects, we computed two quadratic contrasts over the *First*, *Second* and *Third* conditions. Neither contrast revealed any activation in the RHC (neither at *p_FWE_*<.05 nor at a more lenient *p_uncorrected_* <.001 threshold), indicating the repetition effect in the RHC was predominantly linear in nature. In fact, none of the regions exhibiting a linear decrease over repetitions were evident in the quadratic contrasts. On the other hand, two regions exhibiting a *Third*>*Second*>*First* effect also exhibited a quadratic effect (see annotations in Table 3), with a steep increase in activity between *First* and *Second* which then plateaued. Specifically, activity in the right inferior frontal gyrus (*xyz* 58 14 10; see Figure 2B) and right inferior parietal lobule (−58 −46 36) followed this pattern. Note that although a quadratic effect is apparent for the right precuneus in Figure 2B, this effect just failed to reach significance (*p_FWE_* = .06).

To ensure that these changes in signal across repetitions were not the result of non-specific linear time-dependent effects, we computed an additional analysis to control for any non-specific linear time-dependent effects occurring across the time interval separating the first and third future trials. In a random-effects flexible factorial model that included the *First, Second* and *Third* future conditions as well as *time_1_* and *time_2_ Control* conditions (created by pairing control trials separated by a time interval similar to that between the *First* and *Third* future trials), we computed a *repetition* × *condition* interaction analysis to identify regions with significant repetition effects (increases or decreases) for the *Future* but not the *Control* conditions. Importantly, we found that the majority of regions reported in our original *First*>*Second>Third* Future contrast were again evident in this *repetition* × *condition* interaction (although some peak voxels were at slightly different locations in the same cluster; see annotations in Table 3), including the left medial prefrontal cortex, bilateral inferior frontal gyrus, left middle temporal gyrus, anterior RHC and bilateral amygdala (*p_FWE_* <.05). In all these regions, the repetition-related decreases in signal were only evident in the future condition, confirming these effects are not influenced by non-specific linear time-dependent effects (Figure 2A). The only regions in which activation decreases were no longer evident in this interaction analysis were right parahippocampal gyrus, right superior temporal gyrus, and right middle frontal gyrus (see Table 3). Moreover, only one of the regions exhibiting repetition-related increases in the original *Third*>*Second*>*First* contrast, right middle frontal gyrus, was no longer significant in the interaction analysis (see Table 3). For all other regions in the original *Third*>*Second>First* contrast, increases in signal over repetitions were only present for the future condition. Overall, these results confirm that the majority of repetition effects we report cannot be explained by non-specific linear time effects.

Additionally, as we found a significant decrease in reaction time over repetitions, we wanted to ensure that this decrease in activation in several core regions was not due simply to a decrease in task difficulty. To examine this possibility, we entered reaction times as a parametric modulation regressor, which allowed us to compute the *First*>*Second>Third* and *Third*>*Second>First* contrasts while controlling for reaction time. Importantly, many of the same regions, including the MTL regions remained activated for the contrast of *First*>*Second>Third* even when controlling for reaction time. However, some regions were no longer active at a corrected threshold (*p_FWE_* <.05): left caudate, right superior temporal gyrus, and left middle temporal gyrus; see annotations in Table 3. The same regions were activated for the *Third*>*Second>First* contrast when controlling for reaction time.

We also ran a random-effects parametric modulation analysis to identify regions in which neural activity correlated with reaction time. However, there was very little activation correlated with reaction time, with no voxels surviving a corrected threshold of *p_FWE_* <.05. Even at a very lenient threshold of *p_uncorrected_* <.05, the prefrontal clusters that did emerge were not in regions comprising the core network. Based on these additional analyses, we believe that difficulty, as indexed by reaction time, cannot explain the repetition effects evident in the current study.

### Functional Connectivity Results

**Figure 3:**
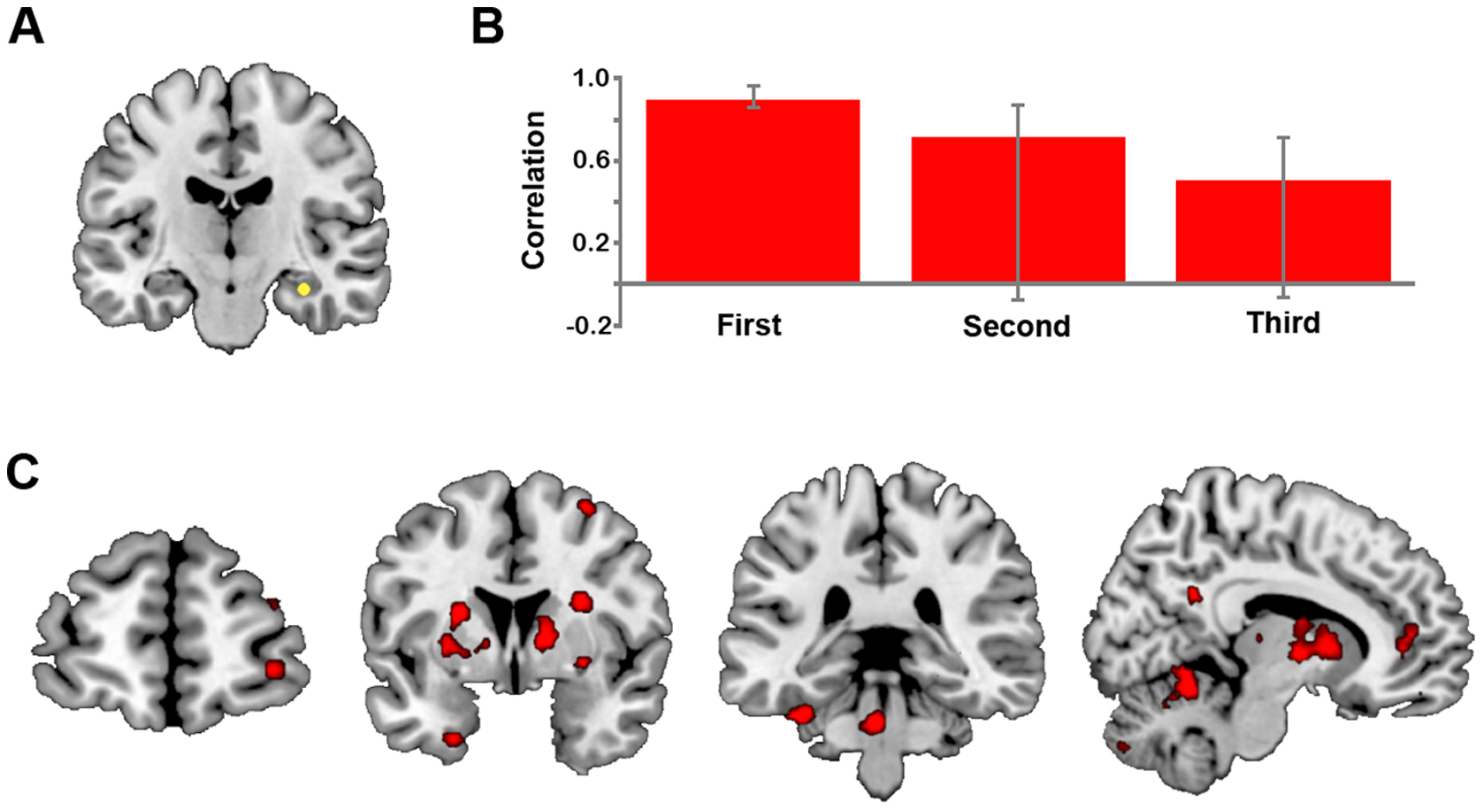
Results from functional connectivity analyses. (A) Location of the RHC seed (*xyz* 32 −16 −18). (B) Average brain scores indicating the strength of correlation between activity in the seed region and the associated whole brain network, with 95% confidence intervals for the *First*, *Second* and *Third* presentations (*p* = .05). (C) Regions which were significantly connected with the RHC seed during *First*, but not during *Second* and *Third* during TR 5, including: right frontopolar cortex (leftmost image), putamen, cerebellum and lingual gyrus (middle images), posterior cingulate cortex, thalamus and right medial frontal gyrus (rightmost image). These data are thresholded using a BSR of 5, which corresponds to *p*<.0001.

We used a seed PLS analysis to examine statistically whether the strength of connectivity of the anterior RHC (*xyz* 32 −16 −18; see Figure 3A) with other brain regions also differs according to repetition. Two *a priori* contrasts were tested: (1) RHC connectivity during *First* but not *Second* and *Third*; and (2) RHC connectivity during *First* and *Second* but not *Third*. This analysis determined that only the first contrast, testing for stronger RHC connectivity during *First* relative to *Second* and *Third,* was significant (*p* = .05), explaining 42.35% of the covariance. This LV indicated that during the *First* simulation, the RHC was strongly connected with a distributed pattern of activity that included many regions associated with simulation, including bilateral medial prefrontal cortex, left MTL regions (parahippocampal and perirhinal cortices) and lingual/fusiform gyrus, and right inferior frontal gyrus, thalamus, and precuneus (see Figure 3C and Table 4). Not only was RHC connectivity reduced during the *Second* and *Third* conditions relative to *First*, but the RHC was not reliably connected to this network during these two repetition conditions, see Figure 3B. Many of the regions exhibiting functional connectivity with the RHC during the first simulation overlapped with, or were adjacent to, regions exhibiting a linear decrease in activity across repetitions (e.g., medial prefrontal cortex, precuneus, putamen), but many regions identified in the two analyses were also distinct.

**Table 4:** Regions showing significant functional connectivity with the right hippocampal seed region.

Brain Region	MNI co-ordinates			BSR
	x	y	z	
L Cerebellum	−6	−74	−30	15.89
R Thalamus	12	−4	6	12.52
R Cerebellum	8	−52	−14	12.09
R Inferior Frontal Gyrus (BA 47)	20	14	−16	11.52
R Medial Frontal Gyrus (BA 10)	20	42	12	11.28
L Parahippocampal Gyrus (BA 36)	−36	−32	−28	11.01
L Cerebellum^†^	−4	−34	−32	10.68
L Putamen	−24	0	18	10.61
R Precuneus (BA 31)	8	−52	30	10.04
L Lingual Gyrus (BA 18)	−22	−76	0	8.87
R Frontopolar Cortex (BA 10)	32	58	−4	8.33
R Middle Frontal Gyrus (BA 6)	30	4	62	8.16
L Perirhinal Cortex (BA 36)	−26	2	−38	8.10
R Thalamus	4	−22	6	7.92
R Middle Frontal Gyrus (BA 46)	50	32	28	7.78
R Superior Frontal Gyrus (BA 9)	18	54	30	7.51
L Cerebellum^†^	−4	−70	−42	7.29
L Middle Frontal Gyrus (BA 9)	−34	32	32	7.01
L Lingual/Fusiform Gyrus (BA 18/19)	−18	−82	−20	6.98
R Putamen	26	16	2	6.93
L Medial Frontal Gyrus (BA 9)	−20	34	32	6.89

during first but not second and third presentation.

*Note:* Only clusters evident during peak time point (TR 5) with a bootstrap ratio greater than ±5 (roughly equivalent to a *p*-value of <.0001) and with a minimum extent of 20 voxels are shown here. BA = Brodmann area; BSR = Bootstrap ratio; L = left; R = right. ^†^Cluster extends bilaterally.

## Discussion

This study extends previous findings of anterior RHC activation during future simulation by demonstrating that activity in this region is stronger during the initial construction of a future event than during repetitions. Right-lateralized activity in the anterior hippocampus was evident during the first simulation trial, and then decreased significantly across repeated imaginings of the same event. The functional connectivity of the anterior RHC with other regions of the core network, including medial and inferior frontal gyrus, was evident during the first, but not the second and third, simulation condition. These findings have important implications with respect to interpreting hippocampal activity (or lack thereof) during simulation: when interpreting such neural effects, it is important to consider whether or not the paradigm requires the active construction of imagined events or reimagining of pre-constructed events during the scanning session. Thus, differential activity for past relative to future events in some previous studies may reflect the influence of the ‘pre-imagination’ sessions in these studies [6], [11], [12].

### Hippocampal Responses to Repetition in Episodic Simulation

Our analyses revealed the presence of repetition-suppression effects in the anterior RHC that were not due to decreases in reaction time or drifts in signal across the repetition interval. The observation that hippocampal activity decreased across repetitions is broadly consistent with other reports that the hippocampus is not consistently active during episodic simulation and may even exhibit a phasic profile of activity [25]. Rather, episodic simulation is likely to be more of a dynamic process, placing varying demands on the hippocampus throughout the generation of a scenario, particularly during the initial phases of simulation, as our results suggest.

One explanation for our results is that RHC activity during the first simulation condition is a novelty response, and that this activity decreases gradually across repetitions with the reducing novelty of simulations across repetitions. Previous work has shown that associative novelty triggers robust hippocampal activation [13], [14], [26], [27]. This idea is also in line with findings from Weiler et al. [15] that the construction of low probability (and more novel) future events invokes increased RHC activation relative to more probable future events. While it might be expected that novelty effects should result in a more steep decline where RHC activity decreases rapidly after initial construction, in line with the idea of novelty detection [28], activity in this region showed a linear decrease with no evidence of a quadratic component. This more linear decrease might suggest that other processes supported by RHC are required less and less, such as binding processes [29]. It is also notable that the functional connectivity of the RHC did follow a marked step-wise pattern: during the first simulation, the RHC was strongly connected with other core network regions, including the left MTL, right inferior frontal gyrus and precuneus, and by the second simulation this connectivity was no longer evident.

Another possibility is that these decreases in activation and connectivity reflect the increasing ease of event construction with repetition. This idea is in line with previous research demonstrating that pre-imagining hypothetical future events increases the fluency of the imagined event, leading to increased plausibility with repetition [30], [31], [32]. It is also possible that reducing demands on detail recombination may have resulted in lowered activity in the hippocampus [10], [14]. One problem with this suggestion is that detail ratings increased across repetitions, and to the extent that increased detail ratings reflect increased constructive processing, it might be expected that hippocampal activity should show a corresponding increase, which it does not. It is also important to note that encoding processes may have also diminished across repetitions. However, unlike other variants of this paradigm [33] that allow a distinction between successful encoding and construction by comparing remembering and forgetting of simulations on a later recall test, we cannot distinguish encoding and constructive processes in this study due to the lack of forgotten trials after three repetitions.

Although it is not possible in this study to distinguish definitively between neural responses to novelty and constructive processes, the finding from the parametric modulation analysis that reaction time did not correlate with activity in medial temporal regions provides some evidence against the interpretation of these repetition effects in terms of increased ease of constructive processes. However, it was somewhat surprising that no regions associated with simulation exhibited correlations with reaction time, which might be expected if reaction time is closely associated with changes in the ease or difficulty of construction processes. While this observation raises some concerns about the use of reaction time as a proxy for difficulty of future event construction, it is noted that some regions were no longer significant in the *First*>*Second*>*Third* analysis once we controlled for reaction time. But nonetheless, while the observed repetition effects cannot be attributed to reaction time effects, they may not necessarily be independent of difficulty. It might also be that some neural regions, such as the hippocampus, respond differently to task difficulty and possibly in ways not adequately captured by reaction time or by a parametric modulation analysis. For example, Summerfield et al. [25] reported that constructing scenes with an increasing number of elements (an increasing difficulty as indicated by ratings) resulted in an overall increase in RHC activation. However, this increase was not linear but phasic. Moreover, the lack of reaction time effects in regions exhibiting repetition effects could also be taken as reflecting processes on a time scale not associated with reaction time, such as novelty effects. Although more fine-grained research is needed to draw strong conclusions, novelty effects may be an important mechanism underlying at least some of the repetition effects reported here. Thus, it remains an important challenge for future research to develop a manipulation or paradigm than can distinguish between novelty and construction of autobiographical future events.

Another important consideration is whether these novelty effects are tied to future simulations specifically, or whether they would be evident for any form of episodic simulation, such as simulations of past or atemporal events [34]. Recent studies on counterfactual episodic simulation, where individuals simulate alternative outcomes to past events, raise the possibility that this effect is not restricted to future simulations, but would extend to other forms of episodic simulation. For instance, De Brigard et al. [35] have shown that episodic memory and episodic counterfactual simulation rely, to a large degree, on the same common pattern of brain activity that is associated with episodic future simulation [2].

Our results suggest that repetition-related decreases in RHC activation and connectivity may occur even when there are some changes to the event representation across repetitions; for related results, see [36]. Although participants rated their repeated simulations as highly consistent (although these ratings may have been somewhat inflated due to the delay between the simulation and post-scan rating phases), there was an incremental increase in detail ratings. We suggest that consistency ratings reflected the maintenance of the gist or core components of the event representation across repetitions, and the small changes in overall event clarity or vividness as indexed by detail ratings were not sufficient to disrupt repetition suppression effects. This point may have important implications for studies using any form of pre-generation of future events.

### Core Network Region Responses to Repetition in Episodic Simulation

Decreases in activity over repetitions were evident in other MTL regions including bilateral amygdala, and were unrelated to reaction time or signal drifts. While amygdala activity is not always evident in future simulation, it has been documented in the two other studies using the recombination paradigm [10], [33]. The randomized recombinations of episodic details probably resulted in a number of uncommon scenarios, and indeed the simulations generated in this paradigm are particularly novel, with low ratings of similarity to previous experiences and thoughts relative to other reports (e.g., [8]). This finding is consistent with studies implicating the bilateral amygdala in the processing of unusual stimuli [37], [38] and novelty detection [28], [39].

A number of extra-MTL regions thought to play an important role in event construction also exhibited a reduction of activity across repetitions that could not be accounted for by reaction time or signal drift. Such regions included the bilateral inferior frontal gyri, an area of prefrontal cortex that is thought to play a role in the generative aspects of future simulation, including semantic generation of event schemas [2], [40]. Interestingly, neither Botzung et al. [11] nor D’Argembeau et al. [12] reported inferior frontal activity, possibly indicative of diminished generative processes following a pre-imagination session. Moreover, our functional connectivity results indicated that during the initial construction, the RHC was functionally connected to the right inferior frontal gyrus, and also to left MTL regions, namely the perirhinal and parahippocampal cortex. The connectivity between MTL regions is consistent with the predictions of the binding of items and context (BIC) model [41], [42], [43], which posits that in order to construct complete representations of episodes, the hippocampus binds item information represented in perirhinal cortex with context information represented in parahippocampal gyrus. It is also possible that connectivity with the fusiform gyrus reflected the integration of person (face) information into the scenario [44]. Together, this connectivity pattern is consistent with the demands of the recombination paradigm, which requires integration of objects and people into particular contexts, and thus suggests this pattern of connectivity may reflect, at least to some extent, the construction demands of the task. This is also broadly consistent with scene construction theory, which poses that event construction involves the binding of multimodal elements into a spatially coherent scene [45], [46].

### Repetition Enhancements in Core Network Regions

A distinct set of prefrontal and parietal regions showed the opposite profile, with activity increasing across repetitions (i.e., ‘repetition enhancement’), including left anterior and right ventrolateral prefrontal cortex, right inferior parietal lobule, right posterior cingulate gyrus and right ventral precuneus. Numerous studies report repetition enhancements in posterior cingulate and ventral precuneus [47], [48], which are thought to reflect source recognition and retrieval [49], [50]. Moreover, left anterior prefrontal cortex (BA 10) has been associated with context retrieval [51], [52], in particular the retrieval of details that have been internally generated [53]. Thus, in the current study, these increases may be a result of retrieving the contextual details comprising previously imagined events, ensuring the consistency of the simulated event across repetitions. Even so, such retrieval processes did not influence levels of MTL activity which steadily decreased across repetitions.

## Summary

Our results demonstrate that the anterior RHC plays an important role in the functional network supporting the initial construction of imagined future events, but that RHC activation and connectivity decreases over repetitions. This finding is important for understanding some incongruent findings reported in the literature concerning hippocampal activation during simulation by confirming that pre-imagining future events has a significant effect on both the activation of the RHC and the broader network supporting the future event simulation. This experiment also provides further support for the idea that generating novel future events particularly enhances the activation of the RHC. Whether such novelty effects are evident for all forms of simulated events (such as counterfactual and atemporal events) remains a question for future research.
